# Potential Role of Selenium in the Treatment of Cancer and Viral Infections

**DOI:** 10.3390/ijms23042215

**Published:** 2022-02-17

**Authors:** Aseel O. Rataan, Sean M. Geary, Yousef Zakharia, Youcef M. Rustum, Aliasger K. Salem

**Affiliations:** 1 Department of Pharmaceutical Sciences and Experimental Therapeutics, College of Pharmacy University of Iowa, Iowa City, IA 52242, USA; aseel-rataan@uiowa.edu (A.O.R.); sean-geary@uiowa.edu (S.M.G.); 2 Department of Internal Medicine, Division of Hematology and Oncology, University of Iowa, Iowa City, IA 52242, USA; yousef-zakharia@uiowa.edu; 3 Holden Comprehensive Cancer Center, University of Iowa, Iowa City, IA 52242, USA; 4 Department of Internal Medicine, Carver College of Medicine, University of Iowa, Iowa City, IA 52242, USA; 5 Roswell Park Comprehensive Cancer Center, Department of Pharmacology & Therapeutics, Buffalo, NY 14203, USA

**Keywords:** selenium, cancer, drug resistance, chemotherapy, viral infection

## Abstract

Selenium has been extensively evaluated clinically as a chemopreventive agent with variable results depending on the type and dose of selenium used. Selenium species are now being therapeutically evaluated as modulators of drug responses rather than as directly cytotoxic agents. In addition, recent data suggest an association between selenium base-line levels in blood and survival of patients with COVID-19. The major focus of this mini review was to summarize: the pathways of selenium metabolism; the results of selenium-based chemopreventive clinical trials; the potential for using selenium metabolites as therapeutic modulators of drug responses in cancer (clear-cell renal-cell carcinoma (ccRCC) in particular); and selenium usage alone or in combination with vaccines in the treatment of patients with COVID-19. Critical therapeutic targets and the potential role of different selenium species, doses, and schedules are discussed.

## 1. Selenium as a Trace Element 

Selenium is a critical microelement that was discovered and isolated for the first time in 1817 by Swedish chemist Jöns Jacob Berzelius [1]. It can be found in both organic and inorganic forms, with selenocysteine, seleno-L-methionine (SLM), and Se-methylselenocysteine (MSC) [2] being the primary organic forms, while selenates (e.g., SeO_4_^2−^, HSeO_4_^−^, H_2_SeO_4_), selenades (e.g., H_2_Se, HSe^−^), and selenites (e.g., SeO_3_^2−^, HSeO_3_^−^, H_2_SeO_3_) are the main inorganic forms [3]. 

### Selenium Forms, Metabolism, and Excretion

**Figure 1:**
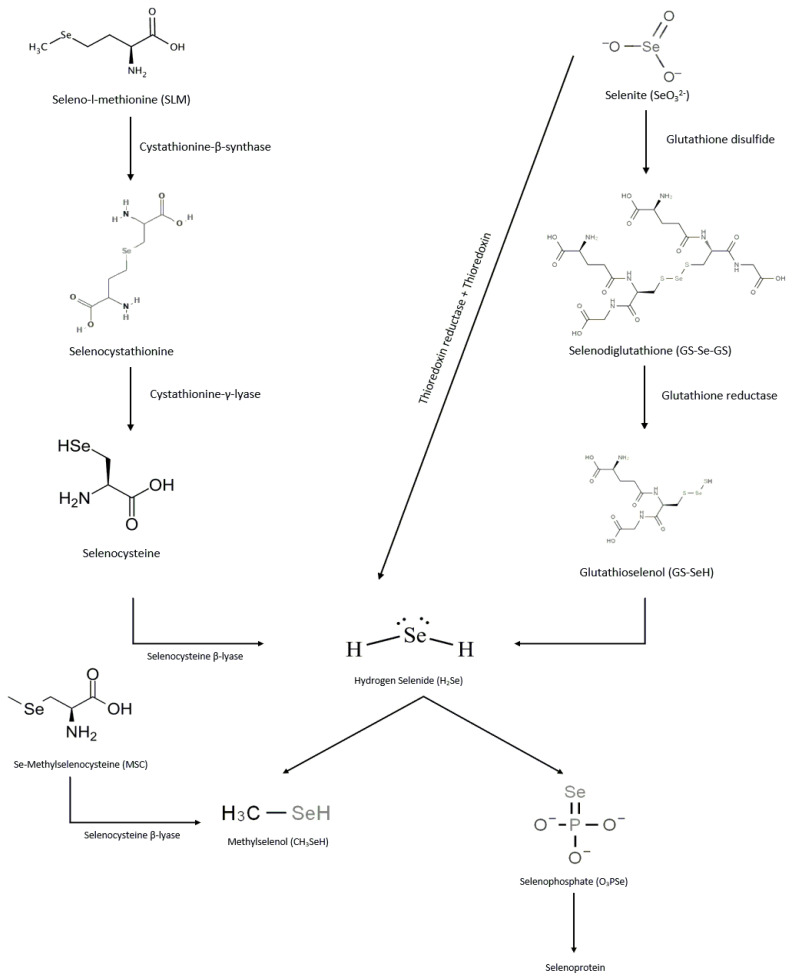
Pathways of selenium species metabolism.

In the human body, selenium metabolism has different pathways as shown in Figure 1. Via cystathionine β-synthase, the organic form of selenium, SLM, is converted to selenocystathionine, which is then sequentially converted to selenocysteine and hydrogen selenide (H_2_Se) by cystathionine γ-lyase and selenocysteine β-lyase, respectively [4]. In contrast to SLM, MSC is directly converted to methylselenol by selenocysteine β-lyase. In the case of selenites, they are either reduced by thioredoxin reductase and thioredoxin to form H_2_Se, or converted to selenodiglutathione by glutathione disulfide, which is then reduced by glutathione reductase to glutathioselenol to produce H_2_Se. H_2_Se can then be methylated to methylselenol or converted to seleno-phosphate for incorporation into selenoproteins during biosynthesis [5]. Methylselenol can be further methylated to dimethylselenol or trimethylselenol. Dimethylselenol can be excreted through exhalation, whereas trimethylselenol is mainly excreted through urine [6]. The conversion of hydrogen selenide to methylselenol is a rate-limiting process that occurs at high selenide concentrations [7]. Based on the differences in the metabolic activation of various selenium molecules, it is likely that MSC offers potential therapeutic advantages over others. In the US, however, only SLM is FDA approved for clinical use [8].

## 2. Selenium as Immune Modulator

Selenium exhibits a wide range of contradictory properties in living organisms. The effect of selenium on the immune system is one example. Selenium has been shown to have an immune-boosting effect and an immune-suppressing effect. Some of the immune-boosting properties of selenium are due, at least partially, to its involvement in enhancing activation and proliferation of B cells (as seen in humans) and/or its ability to promote immune cell differentiation (as seen in murine studies) [9,10]. Deficiencies in selenium have been shown in mice to affect the gut microbiota in such a way as to cause greater susceptibility to infection with Salmonella typhimurium [11], while supplementing humans with low selenium levels, overall, has been shown to augment immune responses and resistance to viral infection [12]. A study conducted by Hoffman et al. demonstrated that selenium supplementation in mice promoted T cell receptor signaling that pushed T cell differentiation toward a Th1 phenotype by increasing interleukin -2 (IL-2) and interferon gamma (INF-γ) production. In contrast, selenium deficiency skewed T cell differentiation toward a Th2 phenotype and depressed activation states, leading to low level T cell receptor signaling. This study also showed that adequate intake of selenium can maintain the balance between Th1 and Th2 [10].

A study conducted in 18 human subjects treated with 200 μg selenium-enriched broccoli daily for three days showed that selenium supplementation resulted in substantially higher levels of both Th1 and Th2 cytokines secreted by peripheral blood mononuclear cells. This study also reported that supplementation with selenium-enriched broccoli resulted in increased plasma selenium levels, while selenoprotein P, which is considered the primary form of selenium storage and transport in the body, remained unchanged [13]. Another study showed that supplementation with selenized yeast increased the percentage of CD4+ T helper cells and reversed tumor-induced CD8+ T cell suppression in EMT6 breast-tumor-bearing mice [14,15].

Conversely, a study conducted by Wang et al. on hens supplemented with three different concentrations of selenium (5 mg/kg, 10 mg/kg, and 15 mg/kg) orally for three time periods (15, 30, and 45 days) found that excessive selenium intake leads to a substantial reduction in the amount of IFN-γ and IL-2 cytokines in both serum and the thymus, as well as a low to moderate incidence in pathological changes in the histology of the thymus tissue, implying a reduction in immunity and an increase in oxidative damage [16].

Collectively, based on the data generated, it is likely that effective modulation of the immune response by selenium may depend on initial selenium status of the individual as well as on the selenium type, dose, and schedule.

## 3. Testing Selenium as a Potential Chemopreventive Agent

The chemopreventive, cancer-promoting, and anti-cancer effects of selenium are three different aspects of selenium’s complicated relationship with cancer. Selenium was prohibited as a food additive by the US FDA for fear of its potential link to cancer as deduced from laboratory animal experiments [17]. However, subsequent studies in 1957 by Klaus Schwartz and Calvin Foltz demonstrated the beneficial effect of selenium intake for humans and mammals by showing that dietary selenium intake protects rats from liver necrosis [1,18]. Selenium was first suggested as a chemopreventive agent in the late 1960s [19] and in the 1990s it was considered a promising cancer preventing agent against different cancer types; however, subsequent clinical trials revealed that selenium did not have chemopreventive properties [20]. One of these trials was the SELECT “Selenium and Vitamin E Cancer Prevention Trial” study. The aim of this study was to determine if selenium, vitamin E, or both could be used as chemopreventive agents against prostate cancer. A total of 35,533 men from the United States, Canada, and Puerto Rico participated in the SELECT study, with all the participants being aged ≥ 50 years of age. Participants were given 200 µg/day selenium (in the form of SLM) with or without 400 international units (IU)/day vitamin E. Unfortunately, after 5.5 years, the results of this study revealed no relationship between selenium supplementation and prostate cancer risk reduction in men with low selenium levels [21]; instead, they discovered that taking selenium supplements raised the high-grade prostate cancer risk in men who had high selenium levels [22]. The lack of a substantial correlation between selenium supplementation and reduced prostate cancer risk was reported after a 1.5-year follow-up period on participants after they stopped taking the supplements [21]. Owing to a lack of effectiveness, an increase in diabetes incidence among selenium-supplemented subjects, and a surplus of selenium, the SELECT study was halted on the recommendation of the Data Safety Monitoring Committee [21]. The controversy over the chemopreventive activity of selenium in various types of cancer can be attributed to at least two factors. One is related to the types of selenium compounds used in the different studies. For example, in the SELECT trial, the type of selenium used was SLM, which showed no chemopreventive activity, whereas in the Nutritional Prevention of Cancer (NPC) trial, selenized yeast was used, which, while mainly containing SLM, also contains MSC. MSC is much more effective as a chemopreventive drug than SLM according to previous preclinical studies [23]. Another contributing factor could be the difference in blood selenium levels of the participants in different studies. For instance, in the SELECT trial, the participants were healthy individuals with normal serum selenium levels, whereas in the NPC trial, the participants had low serum selenium levels. 

Several studies have shown that selenium has a tumor-promoting effect. The NPC trial, for example, found that selenium supplementation (as selenized yeast; 200 µg/day) significantly increased the risk of non-melanoma skin cancer and squamous-cell carcinoma [24]. Another study was conducted on the population of the Reggio Emilia municipality in Italy, who were exposed to 7–9 μg/liter of selenate in tap water from 1975 to 1985. Melanoma incidence was 3.9 times higher in selenium-exposed people than in non-selenium exposed people, according to the findings of this study [25].

## 4. Therapeutic Potential of High Doses of Selenium in Combination with Anticancer Drugs: Preclinical and Clinical Development

**Figure 2:**
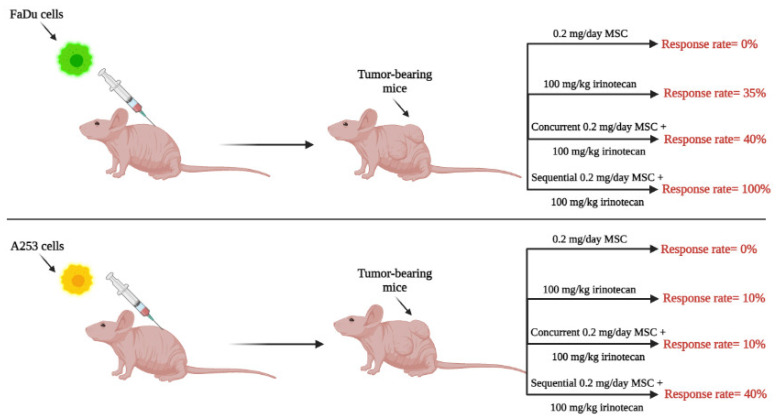
The in vivo response rates of mice bearing tumors treated with 0.2 mg/day MSC, 100 mg/kg irinotecan, concurrently versus sequentially. The concurrent administration of MSC and irinotecan involved administering MSC orally daily for four weeks and irinotecan IV once a week for four weeks, whereas the sequential administration involved administering MSC orally daily for seven days before starting irinotecan, and then starting irinotecan with a weekly dose for four weeks with a daily oral dose of MSC. FaDu and A253 are human squamous cancer cell lines of the head and neck.

Since SLM doses of 200 μg/day (in humans) in prevention trials did not demonstrate a protective benefit [21], the focus of this section is to review therapeutic (rather than prophylactic) data generated with higher doses of SLM or MSC and used in combination with specified chemotherapeutic agents. Using several human tumor xenograft models, our laboratory was the first to demonstrate therapeutic synergy between selenium and anticancer drugs, findings that were dependent on selenium dose and on the sequence of drug administration [26,27,28,29,30]. In general, de novo drug-resistant tumors were found to require higher selenium doses for optimal responses compared with drug-sensitive tumors. The timing of selenium administration when combined with anticancer drugs was determined to be critical, as shown in Figure 2. Utilizing the optimal nontoxic dose of MSC, 10 mg/kg/day and the maximum tolerated dose of irinotecan (100 mg/kg/Wk × 4), optimal therapeutic benefits with high “cure” rates (i.e., complete remission) were only achieved with the sequential combination of MSC, administered orally daily for seven days prior and then concurrent with the intravenous (IV) administration of irinotecan starting on day seven. The demonstration that the administration of the optimal dose of MSC offered selective protection of healthy mouse tissues from toxicities induced by lethal doses of irinotecan allowed the administration of higher irinotecan doses, resulting in further increases in “cure” rates and reversals of resistance [29,31]. In addition, administration of optimal SLM or MSC doses of 0.2 mg/mouse/day × 7 to mice with xenografts of advanced head and neck cancer (human tumors) resulted in stabilization of tumor vasculature and increase in drug delivery of several anticancer agents selectively to tumor tissue [27,32,33]. Collectively, results generated in several xenograft models demonstrated: (1) limited therapeutic efficacy when selenium was administered as a monotherapy; (2) dose-dependent and sequence (of administration)-dependent optimal therapeutic benefit (with high “cure” rate) when selenium was used in combination with certain anticancer agents; (3) stabilization of tumor vasculature by selenium that was dose-dependent and time-dependent; and (4) that selenium provided protection of normal tissues from drug-induced toxicity and thus may offer an approach to mitigate tumor drug resistance by enabling administration of higher drug doses.

Therapeutic results generated in xenografts with the sequential combination of selenium with anticancer drugs provided the rationale to test the hypothesis that administration of the high, nontoxic doses of SLM will increase the efficacy of axitinib (a small molecule that targets VEGFR 1–3) without enhancing toxicity in patients with advanced clear-cell renal-cell carcinoma (ccRCC) (NCT02535533). Eligible patients were treated twice daily with escalating doses of SLM (2500, 3000, and 4000 µg for 14 days, then once daily in combination with 5 mg axitinib twice daily starting day 14 and continuing until disease progression or unacceptable toxicity occurred) [2]. The objective response rate (ORR) of the 31 ccRCC patients treated with one or more cycles of SLM + axitinib was 55% (three complete responses [CR] +14 partial responses [PR]), median progression-free survival (mPFS) = 9.92 months, and median overall survival (mOS) –17.2 months. From the 31 ccRCC patients, five had sarcomatoid morphology and 26 had non-sarcomatoid morphology. One out of five sarcomatoid ccRCC patients demonstrated a PR (20%) [34]. The ORR of the 26 ccRCC patients with non-sarcomatoid morphology and treated with one or more cycles of SLM + axitinib was 62% (3 CR + 13 PR), the mPFS = 17.1 months, and mOS = 25 months. The blood selenium concentrations were dose- and time- dependent. The blood selenium concentrations in the CR patients were 34 µM on day 14 and 129 µM at 7 months, concentrations that were similar to those found to be therapeutically optimal in preclinical studies using tumor xenografts [35]. In contrast, the blood selenium concentrations in PR patients were 20 µM on day 14 and 69 µM at 7 months. In terms of toxicity, grade 3 toxicities included fatigue, hypertension, and weight loss with no grade 4 toxicities or deaths being reported with this combination. Thus, high SLM doses in combination with axitinib in cancer patients are safe and demonstrated encouraging antitumor efficacy. The results also demonstrated that SLM dose timing of the administration of axitinib and the duration of SLM + axitinib are critical determinants for durable outcomes. The encouraging clinical results indicated that accurate translation of the hypothesis based on findings from preclinical models can have significant clinical impact. The ongoing clinical trial is being expanded to include the evaluation of high-dose SLM in combination with axitinib and immune checkpoint inhibitors.

## 5. Molecular Mechanisms of Selenium’s Anti-Cancer Effect and Drug Resistance-Mitigating Ability

It is possible that mechanisms of action of selenium may depend on selenium type, dose, schedule, and tumor profile. In this mini-review, mechanisms associated with high doses of SLM or MSC in the context of ccRCC, head and neck cancer, and lung cancer were discussed.

### 5.1. MicroRNAs-210/155 and Hypoxia-Inducible Factor-1α and -2α

Onco-microRNAs (miRs) and hypoxia-inducible factor 1α and 2α (HIFs) are targets commonly up-regulated in the majority of advanced tumors. Functionally, HIF-1α is reported to regulate glycolysis, whilst HIF-2α regulates genes associated with lipoprotein metabolism and is predominately involved in late stages of cancer [36]. While the list of miRs altered in cancer tissues continue to expand, the oncogenic miRs, miR-210 and miR-155 [37], are ubiquitously altered and co-expressed with HIFs in biopsies, cell lines, and xenograft tumors derived from ccRCC patients [8]. Although miR-210 and miR-155 are induced by hypoxia and considered as hypoxia biomarkers, these miRs are significantly overexpressed in normoxic ccRCC cell lines expressing HIFs and mutated VHL, which is a tumor suppressor gene located on the short arm of chromosome 3 [38,39]. Collectively, these biomarkers regulate the expression levels of many target genes implicated in increased angiogenesis, tumor growth, and drug resistance. 

Recognizing that HIFs and the aforementioned oncogenic miR biomarkers are potential critical therapeutic targets, we hypothesize that agents, such as selenium, that down-regulate these biomarkers may result in selective tumor cell sensitization to subsequent treatment with mechanism-based combinations of biologic and chemotherapeutic agents [30].

Results generated in our laboratory demonstrated that unlike human squamous cell carcinoma of the head and neck, and colorectal carcinoma, HIFs and miRs are constitutively expressed in ccRCC tumor cell lines, xenografts, and primary biopsies [40,41]. Increased tumor angiogenesis and drug resistance may be regulated differentially by HIF1α/2α and by oncogenic miRs, such as miR-155 and miR-210, that regulate pathways independent of HIFs. Thus, agents such as SLM and MSC that target both HIFs should offer greater therapeutic impact than targeting a single HIF phenotype. 

Selenium down-regulates HIFs, leading to the subsequent down-regulation in expression of several genes including those involved in angiogenesis such as vascular endothelial growth factor (VEGF) [42]. The hypoxia response pathway is mediated primarily by HIF-1α/2α, a heterodimeric protein complex that acts as a transcription factor in response to cellular hypoxia. HIF-1 is composed of two subunits: HIF-1α, which is regulated in an oxygen-dependent manner, and HIF-1β, which is constitutively expressed. Under hypoxic conditions, HIFs dimerize with HIF-1β and then this dimer translocates to the nucleus where it binds to a cis-regulatory element on the DNA. This binding will result in the transcription of genes (e.g., VEGF) that will help cells adapt to the shortage of O_2_ [43]. HIF-2α is a homolog to HIF-1α that is also regulated in an oxygen-dependent manner, is structurally similar to HIF-1α, and similarly binds to DNA but has a different transactivating domain [44]. Cancer cells produce HIF in response to hypoxia in order to generate more VEGF (and other growth factors) that promote angiogenesis, thereby facilitating the delivery of much-needed nutrients and O_2_ to the growing tumor. This makes HIF a potential target for cancer therapy [45]. 

In brief, miRNA-210/155 and HIF-1α and 2α are considered master regulators of target genes implicated in increased tumor angiogenesis, metastasis, DNA repair, unstable tumor microenvironment (TME), and multidrug resistance. Down-regulation of these biomarkers by a defined SLM or MSC dose and sequence was associated with multidrug sensitization of tumor cells in several xenografts [30]. 

### 5.2. DNA Repair as a Selenium Target

**Figure 3:**
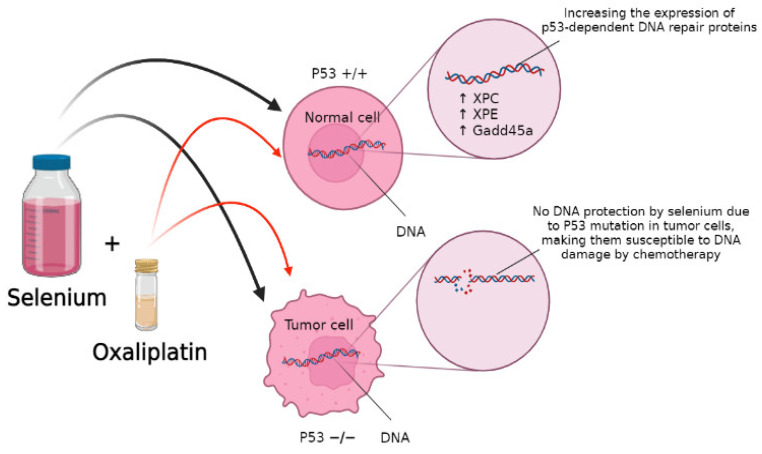
Selenium exposure caused elevation in the expression of XPC, p48XPE, and Gadd45a, which are p53-dependent DNA repair proteins.

Selenium also helps with DNA repair in response to DNA-damaging agents, which improves the effectiveness of chemotherapeutic agents by protecting normal cells from their toxicity. One study with wild-type (WT) and p53 −/− mouse embryonic fibroblasts (MEF) pre-treated with 10 μmol/L of SLM for 15 h followed by cisplatin or oxaliplatin found that selenium protected WT-MEF from DNA damage in a p53-dependent manner by increasing the expression of p53-dependent DNA repair proteins such as XPC, XPE, and Gadd45a, as shown in the Figure 3. Thus, cells lacking p53, such as tumor cells, did not receive the same protection [46]. 

### 5.3. Nrf2 as a Selenium Target 

Nuclear factor erythroid 2-related factor 2 (Nrf2) is emerging as a critical transcription factor that is epigenetically, molecularly, and metabolically regulated [47]. Nrf2 plays dual roles in that it can protect normal tissues against oxidative damage and can act as an oncogenic protein in tumor tissue [48]. Data generated in xenografts demonstrate that a defined dose and schedule of selenium down-regulates and up-regulates Nrf2 in tumor tissue and normal tissue, respectively [30,49,50]. Similar to xenografts, Nrf2 is differentially up-regulated in ccRCC tumors compared with their normal tissue counterpart. Upregulation of Nrf2 in normal tissues and down-regulation in tumor tissues resulted in selective enhancement of anti-cancer drug activity. These differential effects were associated with selective sensitization of tumor tissues to subsequent treatment with chemotherapy. The documented protection of normal tissues from drug-induced toxicity by selenium may be due in part to the activation of Nrf2, resulting in diminished levels of reactive oxygen species, activation of PHDs, which are enzymes that regulates the degradation of HIFs in response to oxygen availability (resulting in enhanced HIF hydroxylation and degradation), and activation of the DNA-repair genes (resulting in enhanced DNA repair involved in tumor cell chemo- and radio-resistance via the Nrf2/Kelch-like ECH-associated protein 1 (Nrf2/Keap1) pathway) [51,52]. This pathway has been found to play a variety of roles in different cancer types. According to some studies, this signaling pathway promotes redox homeostasis and has anti-tumor properties [52]. Overactivation of Nrf2 causes cancer cell survival by inhibiting apoptosis and metabolic reprogramming, according to other studies. Solis et al. discovered that constitutive overexpression of Nrf2 is linked to a poor prognosis for patients with various types of cancer [53].

The resistance of cancer cells to chemotherapy or radiotherapy is a significant issue in the treatment of cancer patients. Multidrug resistance proteins (MRPs) can prevent the accumulation of chemotherapeutic drugs within tumor cells and therefore contribute to drug resistance. Overactivation of Nrf2 has been shown to increase the expression of MRPs, consequently decreasing the effectiveness of chemotherapy [54,55].

Additionally, Nrf2 activation can promote hypoxia resistance, and, when overexpressed (or overactivated) in tumor cells, Nrf2 can promote chemotherapy resistance. Knocking down Nrf2 resulted in suppressed tumor growth in a colon tumor model (mouse xenograft with human colon cancer), with inhibition of angiogenesis being the most likely contributing factor. The inhibition of hypoxia-induced activation of HIF-1α and VEGF by knocking down Nrf2 suppresses angiogenesis, demonstrating a crosstalk mechanism between Nrf2 and HIF-1α in angiogenesis [56]. 

It was also discovered that in vitro hypoxia/reoxygenation activates Nrf2 and thus upregulates peroxiredoxin-1 (Prx1). Keap1 mutations have been found to cause constitutive activation of Nrf2, which results in the loss of tumor suppressor function of Nrf1. Prx1 is a peroxide detoxifying enzyme with a 2-Cys conserved cysteine residue, but it is highly susceptible to inactivation by reactive oxygen species [57]. Under conditions of cellular stress, over oxidation of the 2-Cys residue causes Prx1 to change structurally and functionally from a peroxide detoxifying enzyme to a molecular chaperone, altering the activity of proteins that control cell growth and survival [58,59]. Recent studies by Kim et al. demonstrated that Prx1 increases the clonogenic survival of irradiated lung cancer cell lines and reduces radiation-induced JNK signaling and apoptosis independent of its antioxidant activity, which may explain the chemo- and radio-resistance of various types of cancers expressing high levels of Prx1 [49].

Selenium was shown to reduce drug detoxification and increase cytotoxic effects of anti-cancer drugs in tumor cells through suppression of the Nrf2/Prx1 pathway, while increasing drug detoxification and decreasing cytotoxicity of anti-cancer drugs toward normal tissues by maintaining redox homeostasis. These opposing effects of selenium depend on cell status. The reactive cysteine moiety of redox-sensitive proteins is more vulnerable to thiol modification by selenium in the hypoxic environment of cancer cells, resulting in Nrf2/Prx1 pathway suppression. While under normoxic conditions, this cysteine residue will not be susceptible to selenium modification [28,29,50]. 

One study involving C57BL/6J mice treated with: (1) saline, (2) 15 mg/kg IP doxorubicin, (3) 15 mg/kg IP doxorubicin + 0.2 mg selenium/kg, or (4) 15 mg/kg IP doxorubicin + 0.2 mg selenium/kg + 30 mg/kg IP ML385 (an Nrf2 inhibitor) showed that selenium supplementation attenuated the cardiotoxic effects of doxorubicin by decreasing oxidative stress and inflammation through Nrf2 pathway activation [60]. Another study by Kwon et al. showed that selenium and niacin attenuated sepsis-induced lung injury. In this study, rats were treated with: (1) vehicle, (2) clinically relevant doses of selenium (60 μg/kg), (3) niacin (360 mg/kg), or (4) selenium and niacin in combination. The results showed that the combination of niacin and selenium reduced the reactive oxygen species generated by sepsis and diminished the resultant lung injury by upregulating Nrf2 signaling [61]. 

## 6. Potential Use of Selenium for the Treatment of Viral Infections

Selenium is an essential trace element obtained from diet. Deficiency in blood selenium concentrations has been associated with higher cancer incidence and mortality within countries such as China and India [62]. Additionally, a strong link between selenium deficiency and susceptibility to a variety of viral infections all over the world has been shown. Many researchers have looked at the significance of various types of selenium compounds in preventing enveloped RNA virus infectivity. Keshan disease, caused by the coxsackievirus B3, first appeared in northeast China and showed seasonal variation. When the population was fed with selenium, the incidence of Keshan disease dropped drastically [63], indicating an association between selenium levels and the incidence of the disease. The HIV-1 virus is another example. A 3.5-year study in Miami, Florida looked at 125 HIV-1-seropositive drug-using men and women to see if there was a link between serum selenium levels and death. It was discovered that serum selenium levels and HIV-1 virus mortality had an inverse relationship [64]. Furthermore, it was shown that sodium selenite promotes the proliferation of natural killer cells and suppresses thioredoxin reductase activity, lowering the infectivity of the Ebola virus and other enveloped RNA viruses [65,66]. Recently, research on the relationship between serum selenium levels and COVID-19 cure rates and deaths has been conducted during the period of the COVID-19 pandemic. The mean serum selenium level in Germany was 84 ng/mL in the control group; however, as shown in Table 1, mean serum selenium levels in COVID-19 patients were significantly lower (51 ng/mL) [67]. In India, the control group had mean serum selenium levels of 79 ng/mL, which was significantly higher than that of COVID-19 patients, as illustrated in Table 1 [68,69,70,71]. Using 70 ng/mL as the cut off for defining low selenium levels, 43% of patients with COVID-19 in India had low selenium serum levels while only 20% of controls had low selenium serum levels [68]. Moghaddam et al. reported that 65% of deaths associated with COVID-19 involved patients with low serum selenium levels compared with 39% of patients who survived having low serum levels [67]. Rayman et al. reported a link between COVID-19 cure rate and regional selenium status in China [62,72]. The COVID-19 cure rate was significantly associated with selenium status, as measured by the amount of selenium in hair, in 17 cities outside of Hubei. The researchers speculated whether, given the history of viral infections associated with selenium deficiency, the appearance of COVID-19 in China could possibly be linked to the belt of selenium deficiency that runs from the north-east to the south-west of the country.

All the COVID-19 data presented here suggest that base-line blood levels of selenium are influenced by diet and may serve a rationale for clinically evaluating selenium supplementation of COVID-19 patients treated with vaccines [71].

## 7. Conclusions and Future Strategies

The interactive communication between tumor cells and their surrounding microenvironment contributes to the biological and molecular heterogeneity that can result in resistance to standard therapies such as chemotherapy. The instability of tumor vasculature, a trait of the TME, likely plays a critical role in limiting delivery and distribution of anti-cancer drugs to tumor cells. While the major focus of research has been on tumor cells, significant efforts are underway to characterize and understand the TME as a potential ‘druggable’ therapeutic target. Although significant progress has been made in the treatment of advanced ccRCC, resistance and grade 3 toxicities have been observed in the majority of patients treated with a combination of targeted therapeutics, emphasizing the need to find new ‘druggable’ targets. The overexpression of pro-angiogenic and drug resistant biomarkers, HIF-1α and -2α, miRNA-210/-155, and Nrf2, characterize the unique histologic and molecular profile of ccRCC tumors. Small molecule inhibitors of HIF-2α are under clinical development with promising efficacy in patients with advanced ccRCC; however, ccRCC tumors express both HIF-1α and -2α, and HIF-1α regulates independent and overlapping target genes with HIF-2α. Hence, targeting a single biomarker may not be sufficient to achieve a durable response or cure. The overexpression of these biomarkers provided the rationale and the unmet need to identify small molecules that directly or indirectly target the overexpressed biomarkers. Using several xenograft models, our laboratory was the first to demonstrate that both HIFs and miRNA-210/-155 are selenium targets by a defined dose and schedule of specific types of selenium-containing molecules in combination with anticancer agents. Down-regulation of these biomarkers was associated with complementary effects: 1. normalization of tumor vasculature resulting in enhanced drug delivery to tumor cells; and 2. providing a time window for the administration of anticancer therapeutics. Therapeutic synergy was documented only when anticancer therapeutics were administered at the time when optimal down-regulation of these biomarkers and stabilization of tumor vasculature by selenium were achieved. Based on these findings, the selenium administration time with anticancer agents should be designed in such a way that the maximum down-regulation of these targets is achieved. If this new and novel approach is successful, it could lead to more than incremental improvements in the treatment of ccRCC.

## Figures and Tables

**Table 1:** The mean serum selenium levels (ng/mL) in COVID-19 patients in India and Germany.

Country	India		Germany	
	Control Group	COVID-19 Group	Control Group	COVID-19 Group
Mean serum selenium level (ng/mL)	79	69	84	51
